# Pharmaceutically Active Microbial AhR Agonists as Innovative Biodrugs in Inflammation

**DOI:** 10.3390/ph15030336

**Published:** 2022-03-10

**Authors:** Matteo Puccetti, Marilena Pariano, Claudio Costantini, Stefano Giovagnoli, Maurizio Ricci

**Affiliations:** 1 Department of Pharmaceutical Sciences, University of Perugia, 06123 Perugia, Italy; stefano.giovagnoli@unipg.it (S.G.); maurizio.ricci@unipg.it (M.R.); 2 Department of Medicine and Surgery, University of Perugia, 06132 Perugia, Italy; marilena.pariano@gmail.com (M.P.); claudio.costantini@unipg.it (C.C.)

**Keywords:** indole-3-aldehyde, aryl hydrocarbon receptor, drug delivery

## Abstract

Alterations of the microbiome occur in inflammatory and autoimmune diseases, a finding consistent with the role of the microbiome in the maintenance of the immune system homeostasis. In this regard, L-tryptophan (Trp) metabolites, of both host and microbial origin, act as important regulators of host–microbial symbiosis by acting as aryl hydrocarbon receptor (AhR) ligands. The intestinal and respiratory barriers are very sensitive to AhR activity, suggesting that AhR modulation could be a therapeutic option to maintain the integrity of the epithelial barrier, which has substantial implications for health even beyond the mucosal site. A number of studies have highlighted the capacity of AhR to respond to indoles and indolyl metabolites, thus positioning AhR as a candidate indole receptor. However, the context-and ligand-dependent activity of AhR requires one to resort to suitable biopharmaceutical formulations to enable site-specific drug delivery in order to achieve therapeutic effectiveness, decrease unwanted toxicities and prevent off-target effects. In this review, we highlight the dual activity of the microbial metabolite indole-3-aldehyde at the host–microbe interface and its ability to orchestrate host pathophysiology and microbial symbiosis and discuss how its proper clinical development may turn into a valuable therapeutic strategy in local and distant inflammatory diseases.

## 1. Microbiota and Inflammation

Immune homeostasis is maintained by a precise balance between effector immune cells and regulatory immune cells. Chronic deviations from immune homeostasis can promote the development and propagation of inflammatory diseases, i.e., autoinflammatory and autoimmune diseases, caused by dysregulation in the innate and adaptive immune system (respectively), transplant rejection, etc. Autoinflammatory and autoimmune diseases are clinically heterogeneous diseases with an estimated incidence of approximately 3–5% worldwide [1]. However, it is becoming clear that the immune system and inflammatory processes are involved in a wide variety of diseases that collectively represent the leading causes of disability and mortality worldwide [2]. More than 50% of all deaths are attributable to inflammation-related diseases such as ischemic heart disease, stroke, cancer, diabetes mellitus, chronic kidney disease, non-alcoholic fatty liver disease and autoimmune and neurodegenerative conditions. Evidence is emerging that the risk of developing chronic inflammation can be traced back to early development, and its effects are now known to persist throughout one’s life span to affect adulthood health and risk of mortality [3]. In recent years, the emerging concept that the eukaryotic host and its microbiota exist as a complex meta-organism termed the “holobiont”, jointly regulating multiple aspects of mammalian physiology, including immune system development [4,5], has provided the foundation for the plausible role of the gut microbiota in immune-mediated pathologies [6,7,8]. As a matter of fact, the role of the microbiota in the development of several immune cells, including T helper (Th)1, Th2, Th17 and regulatory T (Treg) cells, is well recognized [9,10]. Organisms such as *F. prausnitzii* and *Clostridium* spp. have a role in directing Treg cells and their response, whereas autoantibodies directed against the cell wall mannan of the yeast *Saccharomyces cerevisiae*, a ubiquitous commensal microorganism, were detected in several autoimmune diseases (i.e., rheumatoid arthritis, systemic lupus erythematosus and anti-phospholipid syndrome) [11,12]. Thus, there is much interest in linking gut microbiota changes to the host immune response in immune-mediated pathologies essentially characterized by aberrant immune responses or a loss of immune tolerance. 

Consistent patterns of dysbiosis between multiple immune-mediated diseases have been observed, as well as microbial signatures, including taxonomic biomarkers that are associated with a particular disease or group of diseases [7,13]. Considering the numerous environmental and genetic risk factors that are known to influence inflammatory pathologies and autoimmune diseases, it is not surprising that some taxa demonstrate varying degrees of differences in abundance. For example, *Actinomyces* and *Eggerthella* spp., both of the Actinobacteria phylum, have been implicated in inflammatory conditions despite historical classification as commensal microorganisms [7]. Crohn’s disease patients, following therapy with enteral nutrition or anti-TNFα antibodies, had decreased *Actinomyces* spp., whereas *Lactococcus* and *Roseburia*, considered to be potential markers of health due to their butyrate-producing and anti-inflammatory properties [14], were increased [7]. Indeed, the depletion of some taxa of the phylum Firmicutes, with known anti-inflammatory properties, and the outgrowth of the phyla Proteobacteria, mainly Enterobacteriaceae, have been linked to human inflammatory bowel diseases, non-alcoholic steatohepatitis and obesity [15,16]. Consistent with the bidirectional communication between the central nervous system and gut—referred to as the “gut microbiota–brain axis”—patients affected by MS exhibit a decrease in the percentage of several Bacteroides, Faecalibacterium and short chain fatty acids (SCFAs)-producing bacteria and an increase in *Methanobrevibacter*, Enterobacteriaceae and *Akkermansia* [17,18]. An association between the gut and skin microbiota composition, immune-mediated skin diseases and the beneficial effect of probiotics in these pathologies has recently also been described [19]. Besides direct inflammatory effects at the skin barrier, microbiota may contribute to the pathogenesis of skin autoimmune diseases by metabolites, recalling immune cell responses and the permeation of antigens to the subepidermal space. Thus, skin and gut barrier dysfunction may represent a common pathophysiologic process allowing microbiota or its particles to promote autoimmune diseases at barrier surfaces [20]. 

Despite the plethora of published data, further research into these microorganisms and their associated functions within the host are needed in order to establish any causality in disease pathogenesis and future therapeutic potential. However, the recent move from descriptive microbiota census analyses to cause-and-effect studies through the generation of high-throughput multi-omics data has offered opportunities to gain insights into the mechanistic understanding of how the gut microbiota affects host immunity and inflammation.

## 2. Metabolites: Messengers between the Microbiota and the Immune System

Among the molecular mechanisms by which host/microbial symbiosis and tissue homeostasis is maintained, the involvement of specific bacterial-derived metabolites has been highlighted. Gut microbial metabolites exert far-reaching influences on the host. They regulate the immune system through histone deacetylases, receptors and/or metabolic integration [21,22]. Thus, by complementing the metabolic capacities of the host, shaping immune system development and activity and regulating the composition and function of the microbiota, it has become increasingly apparent that the microbiota and its metabolites are important orchestrators of host physiology and pathophysiology [4,5]. Among the most abundant molecules produced by gut bacteria are SCFAs, which are known to control multiple aspects of local and distal immunity and metabolism [23]. Other metabolites contribute to the orchestration of immune system maturation at the tissue level by regulating the balance between pro-inflammatory and anti-inflammatory immune responses, such as by the vitamin A lipid metabolite retinoic acid, vitamin D and tryptophan (Trp) metabolites [24]. It was shown that protein malnutrition (and specifically Trp depletion) alters the severity of intestinal inflammation [25]. Angiotensin-converting enzyme 2 (ACE2) controls the expression of the neutral amino acid transporter in the intestine. *Ace2^−/−^* mice featured altered gut microbial composition, and these mice develop severe colitis after the induction of epithelial damage. Transplantation of the gut microbiota to germ-free mice transfers the inflammatory phenotype and susceptibility to colitis, while a Trp-rich diet reverses microbial composition in this model [25]. Similar to *Ace2^−/−^* mice, conditions of Trp deficiency, such as mice lacking the enzyme indoleamine 2,3-dioxygenase (IDO1) (see below), displayed altered microbial composition as well as aberrations in the metabolic pathways of both the microbiota and the host [26]. Of great importance, *Ido1^−/−^* mice showed the blooming of Lactobacilli that use Trp as an energy source to produce ligands of the aryl hydrocarbon receptor (AhR) [26], a multitasking receptor in the immune system [27].

## 3. Microbial Indoles

**Figure 1:**
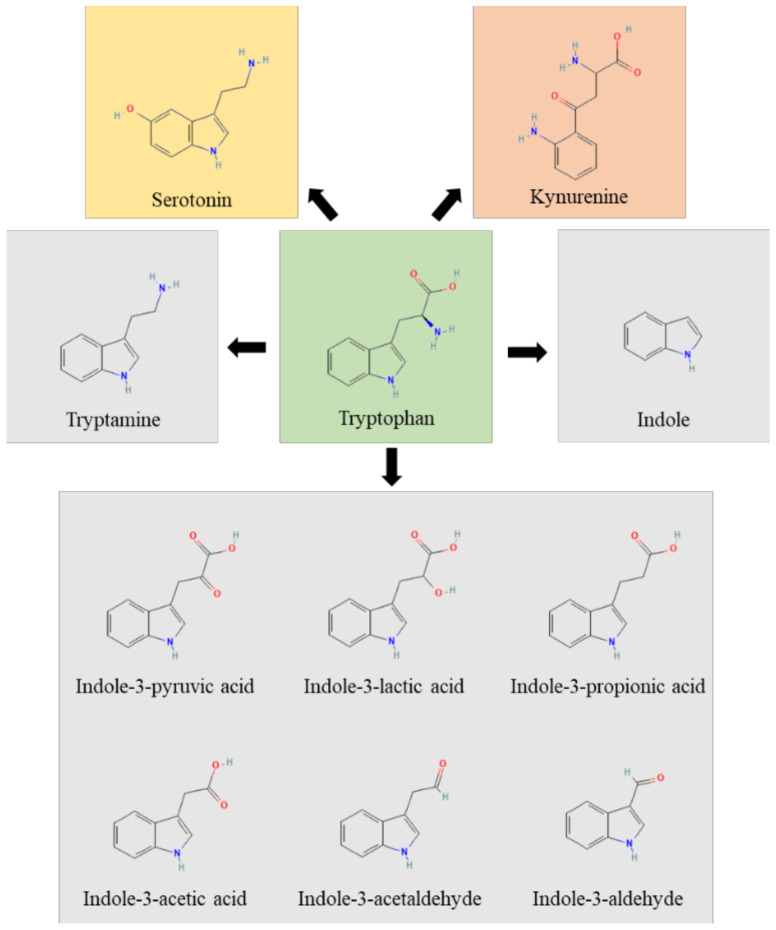
The figure represents the Trp (green box) metabolic pathways of host (yellow and rose boxes) and microbial (grey boxes) origin.

Although some commensal bacterial species can generate Trp de novo, Trp is an essential amino acid, and most Trp found in the human gut is diet-derived. Dietary Trp enters the intestinal tract and reaches its highest relative concentration in the distal colon, where most proteolytic metabolism occurs [28]. Despite the fact that adults’ ingestion of Trp is far above the recommended amount (3.5–6 mg/kg/day), circulating and stored Trp concentrations are the lowest among all amino acids, indicating intense metabolic pathways. Trp-derived AhR agonists are generated through complex biochemical pathways catalyzed by commensal and host enzymes [26,29,30,31,32] (Figure 1). 

In humans, the Trp pathways include: the kynurenine pathway, the indole pathway and the serotonin pathway. The majority of Trp is metabolized by the host through the Kyn pathway, with a small proportion of Trp (estimated to be about 5% of that not used for protein synthesis) being converted into serotonin by enterochromaffin cells. The enzymes indoleamine 2, 3-dioxygenase (encoded by IDO1 or IDO2) and Trp 2,3- dioxygenase (encoded by TDO2) perform a rate-limiting step in the conversion of Trp to Kyn and its downstream metabolites, which ultimately include quinolinic acid, kynurenic acid, NADH and niacin [28]. IDO1 is widely expressed, is highly responsive to inflammation and limits Trp availability, thus affecting pathogen growth and switching T-cell activation to T-cell tolerance [33]. Approximately 4–6% of Trp is metabolized to indole, indican, tryptamine and skatole, as well as indole acid derivatives by the gut microbiota. Indeed, Ampicillin-sensitive, Vancomycin-resistant bacteria generate AhR agonists from dietary Trp [26]. However, the bacterial synthesis of indole compounds occurs via different metabolic pathways mainly involving the activity of tryptophanase, aromatic amino acid aminotransferases and tryptophan deaminases generating, among others, the indole-3-aldehyde (3-IAld) [31,34]. Thus, the gastrointestinal tract harbors numerous species with the capacity to synthesize indole and indole-containing compounds that, by affecting host immune reactivity, epithelial barrier function and pathogen colonization, act as a possible link to microbiota dysbiosis. The production of indoles may be a general property of eubacteria, and the coevolution of indole-producing bacteria with animals over the last ∼500 My may explain how indoles coordinate responses to a myriad of stressors in such diverse organisms. The evolutionary benefit provided to the microbes generating these molecules is unclear, although indole production has been shown to be beneficial for bacterial quorum sensing, motility, antibiotic resistance, biofilm production, and defense against non-indole producers [35]. Of great importance, microbial indoles have been shown to be beneficial to the host by acting as biologically active signaling molecules recognized by host xenobiotic receptors, such as the aryl hydrocarbon receptor (AhR) and the pregnane X receptor (PXR) [32,36,37,38,39]. This dual activity at the host–microbe interface qualifies indoles as unique orchestrators of host pathophysiology and regulators of microbial symbiosis. 

## 4. Therapeutic Targeting of AhR

Ample evidence suggests that microbiota-derived AhR or PXR ligands not only contribute to gastrointestinal homeostasis [40] but also to the etiology of cardiovascular, metabolic and psychiatric diseases [41]. This implicates that the therapeutic targeting of xenobiotic receptors could be of great medical and pharmaceutical interest. Given that intestinal microbes produce Trp metabolites that can signal to the host outside of the intestine, dietary intervention and prebiotics or probiotics to increase beneficial Trp metabolite production would be attractive alternative therapies. However, although Trp feeding was shown to be protective in murine models of colitis [26,42], successful clinical trials or interventions that manipulate Trp or its microbial derivatives to benefit human disease have not yet developed. Despite the availability of thousands of structurally diverse ligands of AhR and PXR, the bottleneck in targeting these receptors is that their functional activity is both context- and ligand-dependent [43]. For instance, the ligand-specific modulation of the AhR signaling pathway has been suggested to result from ligand-dependent changes in the AhR, which could allow it to interact with different nuclear factors or dimerization partners and different coactivators [44]. Thus, AhR function is not necessarily equal to that of AhR ligands in overall immune responses. Nevertheless, the identification of AhR ligands and their positive health effect and beneficial pharmaceutical properties has stimulated studies aimed at developing drugs for various tumors, immune and inflammatory diseases. Various natural products capable of activating AhR for anti-inflammatory effects have been described [45]. However, most of them have inadequate ADME (absorption, distribution, metabolism and excretion) with poor oral bioavailability, and the pharmacologically active doses are always very high. Moreover, the patentability of the natural compounds also limits their development as drugs. Thus, more tailored AhR agonists are desirable. Given the context-and ligand-dependent activity of most xenobiotic receptors, including AhR, this requires one to resort to suitable biopharmaceutical formulations to enable site-specific drug delivery of the appropriate ligand in order to achieve therapeutic efficacy, decrease unwanted toxicities and prevent off-target effects. Over the past two decades, pharmaceutical and medicine systems have experienced tremendous change and advancement in the transition from conventional to novel drug delivery systems. Different types of carriers and their physiochemical and biological characteristics have made therapeutic delivery safer and more efficient. Categorized by the route of administration, the oral, nasal and pulmonary routes have gained importance over the years as alternatives to the parenteral route, especially for peptide and protein therapeutics [46]. Preclinical studies with 3-IAld demonstrate how appropriate drug delivery technologies may convert promising AhR ligands into pharmaceutically active oral and pulmonary compounds (see below).

## 5. Indoles Therapeutics in Inflammatory Diseases: The Case of 3-IAld

**Figure 2:**
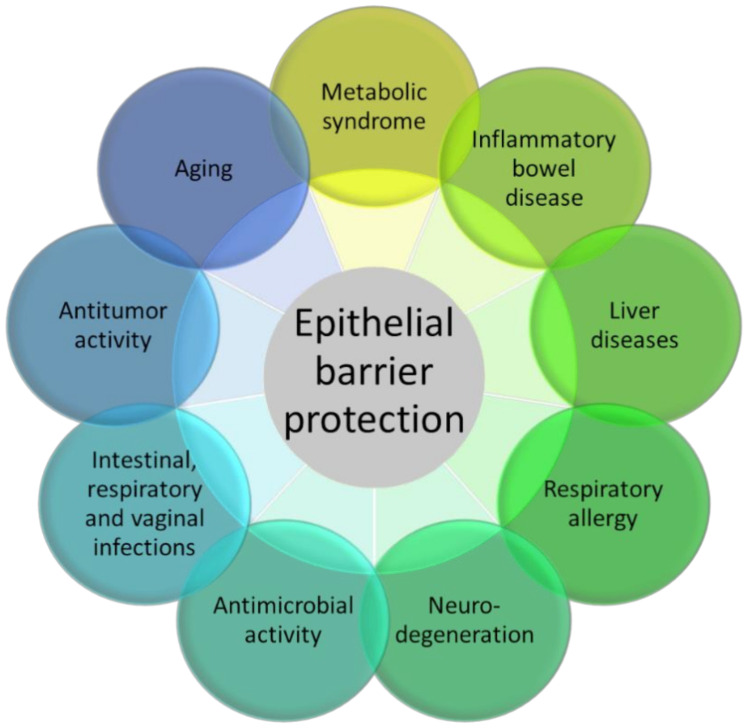
The figure represents the many experimental pathology models in which 3-IAld has shown beneficial activity, essentially moving from its epithelial barrier protection function.

3-IAld is abundantly produced by *Lactobacillus reuteri* in condition of Trp availability via the enzyme aromatic amino acid aminotransferase [26]. 3-IAld triggers the host xenobiotic response mediated by AhR [26] and does not seem to act as a ligand of PXR [47]. 3-IAld has been shown to displace the high-affinity AhR ligand FICZ in a competitive radio-ligand binding assay and to promote the nuclear translocation of AhR [32]. Pharmacokinetics analysis has revealed the rapid metabolic conversion of administered 3-IAld [48], potentially classifying it as a Rapidly Metabolized AhR Ligand [49]. In a comparative screening of microbial indoles, 3-IAld displayed a relatively low affinity, potency and efficacy, with indole, indole-3-pyruvate and indole-3-acetamide showing the higher agonistic activity [32,43]. Thus, although not among the most active endogenous ligands of murine and human AhR, 3-IAld appears to act as a putative AhR ligand likely within the range of concentrations found in murine and human gut [32]. One major activity of 3-IAld relies on its ability to enhance the barrier function and the production of antimicrobial peptides at mucosal surfaces (Figure 2). 

The administration of 3-IAld promoted the function of the epithelial barrier and protected diseased mice from gut inflammation by resorting to different mechanisms, ranging from the production of IL-22 [26], IL-10 [50] and type I interferons [51], to the modulation of the expression of IL-10R [52], the preservation of the apical junctional complex [50] and the cross-talk with enteric neurons [53]. This is consistent with the ability of indoles to expand gut intraepithelial T cells [54], postnatally expand CD4–RORγt+ innate lymphoid cells and control the organogenesis of intestinal lymphoid follicles [55] via AhR. In clinical settings, 3-IAld was shown to protect from intestinal damage in graft-versus-host disease in a murine model of allogeneic bone marrow transplantation [51] as well as provide resistance against hematopoietic, gastrointestinal and cerebrovascular injuries caused by ionizing radiation [56]. 3-IAld was also shown to promote intestinal epithelial homeostasis during aging, thus potentially limiting age-dependent systemic inflammation [50].

The beneficial activities of 3-IAld were also evident during infection, such as against *Candida albicans* in the gut [26] and vagina [57] and *Aspergillus fumigatus* in the lung [58], by promoting epithelial barrier integrity and the release of antimicrobial peptides. In line with indoles working not only as trans-kingdom, but also as interspecies signaling molecules [35], 3-IAld may not only influence the host, but it can also act directly on microbes and modify microbial composition at various body sites. Conversely, an alteration in the composition of the microbial communities may change the metabolic profile and affect the levels of 3-IAld. For instance, in Atopic Dermatitis, dysbiotic changes in the skin microbiota have been associated with reduced Trp metabolism leading to 3-IAld [59], thus limiting the AhR-mediated suppression of skin inflammation [27]. 3-IAld also alleviates inflammatory signs in murine encephalomyelitis via an AhR/type I interferon-axis, limiting NF-KB activation in astrocytes [58]. 

Consistent with the requirement of AhR signaling for the maintenance of lung health and the regulation of inflammation through changes in gene expression, cell–cell adhesion, mucin production and cytokine expression [60], it has recently been reported that the targeted delivery of 3-IAld via dry powder inhalation [61] is an efficient strategy to restore immune and microbial homeostasis, in the relative absence of local and systemic inflammatory toxicity, in the inflamed lungs of mice with cystic fibrosis, a multisystem inflammatory disease [62]. Thus, the enhanced delivery to target organs of AhR agonists, such as 3-IAld, may pave the way for the development of safe and effective anti-inflammatory agents in CF. It is of interest, in this regard, that the production of 3-IAld was defective in the oropharynx of hematologic patients at high risk for fungal pneumonia [63], a finding suggesting that replacement therapy with 3-IAld may be of benefit in respiratory inflammation. Along the same reasoning, enteric formulated 3-IAld was able to activate AhR locally and to prevent immunopathologies, intestinal epithelial barrier dysfunction and liver complications in preclinical models of metabolic inflammation and liver injury [48,64]. Finally, 3-IAld formulated for localized delivery in the gut protected mice from intestinal damage via dual action on both the host and the microbe sides in checkpoint-inhibitor-induced gastrointestinal toxicity [65]. It is worth noting here that protection by 3-IAld occurred via dual action on both the host and the microbes sites. Indeed, paralleling the activation of the host AhR/IL-22-dependent pathway, 3-IAld also affected the composition and function of the microbiota such that fecal microbiome transplantation from 3-IAld-treated mice protected against gut inflammation [65].

These studies provide proof-of-concept demonstration that moving past bacterial phylogeny and focusing on bacterial metabolome may lead to a new class of discrete molecules that work at the interface between microbes, and the host immune system may maintain mucosal homeostasis and alleviate inflammatory pathology.

## 6. Conclusions

Despite the fact that the association between inflammation and chronic disease is now widely recognized, how to translate the body of knowledge into effective strategies for the prevention and treatment of pathologic inflammation to improve human health is lagging behind [3]. An important issue in the development of anti-inflammatory drugs is to maintain an equilibrium between resolving the inflammatory pathology without compromising the ability of the immune system to respond to pathogens. The safety of current standard immunosuppressant therapies is hampered by their systemic administration that increases the risks of off-target effects and toxicity associated with the dose required to obtain a therapeutic effect. Clearance by the reticuloendothelial system may also limit their efficacy. Resorting to targeted delivery, which releases the drugs at the specific site of interest, may offer significant advantages in this direction. The identification of microbial metabolites as regulators of immune responses has greatly impacted areas of fundamental importance to both the conceptualization of host–microbiota interactions and its translation to clinical applications. Indeed, metabolites are not only ideally suited as biomarkers for disease development, as their detection may precede the onset of clinically manifest disease symptoms, but also, their causal involvement in the molecular etiology of human disease offers untapped opportunities for drug discovery and the development of new patient-tailored therapies. In addition to resorting to endogenous metabolites by pharmacological or nutritional intervention, we have provided proof-of-concept demonstration that the exploration of a bacterial metabolite such as 3-IAld to support beneficial bacterial–host symbiosis and cooperation may lead to the rational design of effective clinical interventions in human chronic inflammatory conditions in which epithelial barrier disruption and microbial dysbiosis are causally linked. This requires one to resort to suitable biopharmaceutical formulations to develop more localized and specific immunomodulatory approaches, decrease unwanted toxicities and prevent off-target effects.

## Data Availability

Data sharing not applicable.
